# Supplementary material for: Patterns of Intron Gain and Loss in Fungi
Source: PLoS Biol. 2004 Nov 30;2(12):e422. doi: 10.1371/journal.pbio.0020422 (PMC532390; doi:10.1371/journal.pbio.0020422)
Supplement: Table S1 — Also available at http://genes.mit.edu/NielsenEtAl/. (4.3 MB ZIP). [file pbio.0020422.st001.zip › NielsenEtAl/html/924.html]

AN3593.1.NCU09264.1.MG01608.1.FG00493.1


```
 CLUSTAL W (1.82) Multiple Sequence Alignments - Introns Inserted


Sequence 1: NCU09264.1	258 aa
Sequence 2: MG01608.1	225 aa
Sequence 3: FG00493.1	256 aa
Sequence 4: AN3593.1	240 aa
Alignment Length: 263 aa
Number Identitical Residues: 130 aa
Alignment Score (without introns) 6378


MG01608.1 	MSSDIS-----NNDHLVLSDNPDHPANLIPSLCAKFWTLGWVTGTGGG~CSIREN2DLVY
NCU09264.1	MCSPTTENNNNNNDHLVQSSDPNHPANLIPALCAKFWTLGWVTGTGGG~ASIRSD~DLVY
FG00493.1 	MAAEQQ---RLTGDALITSEDPNHPANLIPSLCAKFWTLGWVTGTGGG~CSIRDD2DLVY
AN3593.1  	MSQGLQ---QQNNDHLVQSDDPEHPANLIPELCRKFYNWGWVTGTGGG0TSIRRG~EHIF
          	*.         ..* *: *.:*:******* ** **:. *********  *** . : ::

MG01608.1 	IAPSGVQKELMKAADIYVLSLAAQTASL---RDRVYLRSPPCYKPSQCTPLFLAAFTKRR
NCU09264.1	LAPSGVQKELMKPDDIYVLSLAAQAQSLD-KRQRVYLRSPANYKPSQCTPLFLAAFTKRN
FG00493.1 	IAPSGVQKELMKNTDIYVMALSEQDPNHNKLNQRTYLRSPPCYKPSQCTPLFLAAFTRRG
AN3593.1  	IAPSGVQKELMQPHNIFVLQYPTPKYPP---SERKYIRKPLDLKPSACTPLFLAAFER-G
          	:**********:  :*:*:  .          :* *:*.*   *** ********* :  

MG01608.1 	AGCCIHTHSQWAVLVTLILEAGGGPGGAEDAREFRINNIEQIKGFGKGFEKSGNLGYHDT
NCU09264.1	AGCCIHTHSHWAVLVTLILETQG----AGKDREFMINNIEQIKGFGKGFGKSGNLGYHDT
FG00493.1 	AGCCIHTHSQWAVLVTLLLESQG----PGKDRVFEINNIEQIKGFGRGMNKTGNLGYHDT
AN3593.1  	AGCCIHTHSQWAVLVTLLVEREK-----GPEGCFEISNIEQIKGIPKGPGK-GMLGYFDT
          	*********:*******::*             * *.*******: :*  * * ***.**

MG01608.1 	LRIPVIENTAHEEDLTEFLEEAMDKYPDTYAVLVRRHGV~YVWGDNVHKAKTQCER~---
NCU09264.1	LKIPVIENTAHEEDLTEFLEEAMDKYPDTYAVLVRRHGV~YVWGENVHKAKTMCES2LDY
FG00493.1 	LRIPVIENTPHEEDLTEYLEEAMDKYPDAYAVLVRRHGV~YVWGDNVHKAKTQCES2LDY
AN3593.1  	LRIPIIDNTAFEEDLTGSLEKAMDAYPDTYAVLVRRHGI2YVWGDNVAKAKTQCES~LDY
          	*:**:*:**..*****  **:*** ***:*********: ****:** **** **   . 

MG01608.1 	---------------------------
NCU09264.1	LFQLAVEMKQLGLPWVTDIEPTVPTRK
FG00493.1 	LFQLAVEMKKLGLPWISEVEQIAPQRT
AN3593.1  	LFQLAVEMHKLGIPWVK----------
          	  . : .  . . .  .
```
